# Diploid origins and early genome stabilization in the allotetraploid *Arabidopsis suecica*

**DOI:** 10.1111/nph.70689

**Published:** 2025-11-05

**Authors:** Robin Burns, Anna Glushkevich, Aboli Kulkarni, Uliana K. Kolesnikova, Filip Kolář, Alison Dawn Scott, Polina Yu. Novikova

**Affiliations:** ^1^ Department of Plant Sciences University of Cambridge CB23EA Cambridge UK; ^2^ Department of Chromosome Biology Max Planck Institute for Plant Breeding Research 50829 Cologne Germany; ^3^ Department of Botany Faculty of Science, Charles University 12800 Prague Czech Republic; ^4^ Department of Plant Biotechnology and Bioinformatics Ghent University 9052 Ghent Belgium; ^5^ VIB‐UGent Center for Plant Systems Biology Ghent 9052 Belgium

**Keywords:** adaptation, *Arabidopsis suecica*, genome evolution, mutation, polyploidy, population genetics

## Abstract

- Polyploidization, followed by genome downsizing, is a recurrent evolutionary cycle that dramatically reshapes genome structure. Newly formed polyploids must quickly adjust their cell division machinery to maintain stable chromosome inheritance, while long‐term stabilization involves rediploidization, returning the genome to a diploid‐like state. Here, we investigate the origin and early genome evolution of *Arabidopsis suecica*, a hybrid polyploid derived from *A. thaliana* and *A. arenosa*.
- Leveraging a recent genome assembly for *A. suecica* along with population‐level whole‐genome resequencing of all three species, we identify the closest progenitors to *A. suecica* and use this knowledge to examine genes under positive selection and to assess compensatory dynamics between homeologs carrying loss‐of‐function mutations.
- Our findings show that both parental species were diploid, including the paternal *A. arenosa* progenitor. We identify evidence for *de novo* adaptation to allopolyploidy within the *A. arenosa* sub‐genome of *A. suecica*, including genes involved in homolog pairing and recombination. Although relaxed purifying selection is evident, likely due to the genome‐wide redundancy, we observe functional compensation between homeologous gene pairs in *A. suecica*: When one copy loses function, the other maintains it.
- Together, these findings revise the origin of *A. suecica* and identify early genome stabilization mechanisms, including evidence for meiotic adaptation and mutational buffering through homeologous gene compensation.

## Introduction

Cycles of polyploidy or recurrent whole‐genome duplications (WGDs) have profoundly shaped the genomes of most eukaryotes (Hokamp *et al*., 2003; Aköz & Nordborg, 2019; Van de Peer, 2023). In plants, allopolyploidy, WGD combined with interspecific hybridization, is both widespread (Soltis *et al*., 1993) and associated with important agronomic traits (Chen *et al*., 2020; Dubcovsky & Dvorak, 2007; Li *et al*., 2015). Major crops, including wheat, cotton, *Brassica*, strawberry, peanut, and tobacco, trace their origins to allopolyploid events (Bertioli *et al*., 2019; Feldman & Levy, 2005; Liston *et al*., 2014; Renny‐Byfield & Wendel, 2014; Sierro *et al*., 2024; Staudt, 2009; Nagaharu, 1935). Understanding the evolutionary success of allopolyploids requires resolving the mechanisms that stabilize their genomes, resolving meiotic incompatibilities, and managing genome‐wide genetic redundancy.

The evolutionary fate of allopolyploids depends on the sequence of events that generate them. One key event is whether WGD occurs before or after hybridization because this can influence how readily a stable genome emerges. When WGD follows hybridization, as in *Primula kewensis* (Newton & Pellew, 1929), *Capsella bursa‐pastoris* (Bachmann *et al*., 2021), and experimental F₁ hybrids in *Mimulus* (Wiiliam & Malcolm, 1971) and *Arabidopsis* (Nasrallah *et al*., 2000), the resulting neo‐allotetraploid must adapt *de novo* to coordinate divergent genomes during basic cellular processes. By contrast, if WGD occurs before hybridization through the fusion of 2n gametes, autopolyploid parents can serve as a natural source, since their reduced gametes are equivalent to unreduced gametes from diploids. Consequently, the resulting allopolyploid could inherit alleles already adapted to polyploidy, potentially easing genome stabilization. The extent to which inherited alleles are essential, or whether independent adaptations frequently arise in young allopolyploids, remains unclear. This question has important implications for both extinction risk and subsequent allopolyploid genome evolution.

Meiotic stabilization is one of the most immediate and consequential challenges facing newly formed allopolyploids. The formation of multivalents and improper pairing between homeologous chromosomes can disrupt segregation and compromise fertility (de Carvalho *et al*., 2021; Gonzalo, 2022; Katche *et al*., 2023a, 2023b). While divergence between sub‐genomes can reduce these risks by limiting homeologous recombination (Stebbins, 1947; Soltis *et al*., 1993; Ramsey & Schemske, 2002), it does not eliminate them entirely (Gaeta & Chris Pires, 2010; Zhang *et al*., 2020; Katche *et al*., 2023a, 2023b). Experimental allopolyploids, including synthetic *A. suecica*, often exhibit meiotic defects despite a substantial subgenomic divergence of 11.6% (Burns *et al*., 2021; Chéron *et al*., 2023; Nibau *et al*., 2024).

To achieve stable inheritance, polyploids evolve genetic mechanisms that modify crossover distribution or suppress homeologous recombination. In autopolyploids, this often involves polygenic adaptations in meiotic genes, including genes in the synaptonemal complex (Hollister *et al*., 2012; Yant *et al*., 2013; Bohutínská *et al*., 2021; Bray *et al*., 2024) and kinetochore (Bray *et al*., 2024), yet whether these same meiotic pathways are changed in allopolyploids remains an open question. Stable allopolyploids do appear to have evolved genetic control of sub‐genome‐specific recombination. Notable examples include *Ph1* (*ZIP4*) (Martín *et al*., 2021) and *Ph2* (MSH7‐3D) (Serra *et al*., 2021) loci in allohexaploid wheat, the *PrBn* locus in *Brassica napus* (Liu *et al*., 2006), and *BYS* in natural *A. suecica* (Henry *et al*., 2014), all of which suppress homeologous recombination. In yeast hybrids, the mismatch repair pathway has been shown to limit crossovers between divergent chromosomes, and loss of MSH2 can restore fertility in sterile hybrids (Greig *et al*., 2003). On a transcriptional level, meiotic‐related genes were shown to be upregulated on the *A. thaliana* sub‐genome of *A. suecica* (Burns *et al*., 2021). However, it remains unclear whether meiotic stabilization in young allopolyploids arises primarily through changes in gene expression or protein‐coding sequences, or both.

Polyploid genomes do not remain polyploid indefinitely. Ancient WGDs in the ancestry of most diploids suggest that current polyploids will eventually return to a diploid‐like state through a process known as rediploidization (Van de Peer *et al*., 2017). This transition reshapes genome architecture via gene loss, regulatory divergence, and biased fractionation (Mandáková & Lysak, 2018; Baduel *et al*., 2019; Monnahan *et al*., 2019; Spoelhof *et al*., 2021; Conover & Wendel, 2022; Redmond *et al*., 2023), as seen in both ancient allopolyploids like maize (Sun *et al*., 2023), and in the diversification of distinct morphotypes in *Brassica* (Cheng *et al*., 2014). During rediploidization, relaxed purifying selection in redundant gene copies allows deleterious mutations to accumulate, while functional homeologs compensate for their nonfunctional counterparts, marking early stages of genome downsizing, as observed in *Brachypodium hybridum* (Scarlett *et al*., 2023). Functional compensation between homeologs may reflect a shift in selective constraint, from individual gene copies to gene pairs, suggesting that polyploid genomes are shaped not just by genetic redundancy, but by selection acting to preserve gene functional integrity. Several studies have shown that deleterious mutations, both point mutations and structural variants, accumulate more rapidly in polyploids than in diploids (Paape *et al*., 2018; Conover & Wendel, 2022; Hämälä *et al*., 2024), likely due to masking effects between redundant gene copies. The extent to which this type of mutational buffering between homeologs is shaped by selection, or if it is merely a neutral consequence of genetic redundancy, remains unresolved. Another uncertainty is whether patterns of early gene loss are predictable, based on gene function or the sub‐genome gene location. While genome dominance and biased fractionation have been reported in some allopolyploid species (Schnable *et al*., 2011; Alger & Edger, 2020; Ma *et al*., 2024), they are notably absent in several natural allopolyploids (Burns *et al*., 2021; Scarlett *et al*., 2023; Sun *et al*., 2023; Penin *et al*., 2024). Gene function has been shown to impact gene fractionation rates, with previous studies showing that genes related to genome integrity and organelle function revert to a single copy level faster than genes involved in signaling, transport, and metabolism, likely due to selective constraints acting on gene dosage levels (Li *et al*., 2016).

The allotetraploid *A. suecica*, a post‐glacial hybrid between *A. thaliana* and *A. arenosa*, is an excellent model for studying the early stages of allopolyploid genome evolution. However, the origin of the WGD in *A. suecica*, whether it arose somatically in a diploid hybrid or was inherited via 2× gametes, remains unresolved. If the paternal *A. arenosa* was autotetraploid, WGD likely occurred through the fusion of its reduced gamete (*n* = 2×) with an unreduced *A. thaliana* gamete (2n = 2×), a parsimonious route (Jakobsson *et al*., 2006; Novikova *et al*., 2017). However, recent evidence from sequence similarity of meiotic genes points to a diploid *A. arenosa* parent (Nibau *et al*., 2022). A diploid *A. arenosa* parent would imply WGD likely occurred somatically in a diploid hybrid and that *A. suecica* had to evolve *de novo* solutions to the challenges of polyploidy. Therefore, distinguishing between diploid and tetraploid *A. arenosa* as the paternal progenitor is critical for understanding the origin of WGD in *A. suecica* and for disentangling inherited vs novel mechanisms of meiotic stabilization.

With a high‐quality long‐read genome assembly of *A. suecica* (Burns *et al*., 2021) and expanded sequence data from both parental lineages, including the full diversity of *A. arenosa* cytotypes (1001 Genomes Consortium, 2016; Novikova *et al*., 2016; Monnahan *et al*., 2019; Burns *et al*., 2021; Bohutínská *et al*., 2023b), we address three central hypotheses about the origin and early genome evolution following allopolyploidy in *A. suecica*. First, we ask whether the *A. arenosa* parent of *A. suecica* was diploid or autotetraploid. The paternal *A. arenosa* identity allows us to infer whether WGD likely occurred somatically in a diploid hybrid or was inherited through the hybridization of 2× gametes. Establishing a diploid origin would support the hypothesis that *A. suecica* had to evolve *de novo* solutions to stabilize meiosis and manage genome‐wide redundancy. Second, we test whether adaptation to allopolyploidy in *A. suecica* involves positive selection on genes that regulate meiosis and genome integrity. To do this, we identify genes under positive selection in the *A. suecica* genome and use protein–protein interaction networks to determine whether they represent important functional clusters. Finally, we ask how *A. suecica* manages the mutational load associated with genome‐wide genetic redundancy. By analyzing the distribution of inherited and *de novo* loss‐of‐function (LoF) mutations across sub‐genomes, we test for a negative correlation between damaging variants in homeologous gene pairs. In other words, we ask, when one copy (e.g. from *A. thaliana*) harbors a LoF mutation, does the other (e.g. from *A. arenosa*) remain intact? Evidence for homeolog compensation would suggest that selection operates at the level of gene pairs, potentially buffering young polyploid genomes against the mutational load that accompanies the polyploid‐induced relaxed selective constraint.

## Materials and Methods

### DNA extraction of herbarium samples and sequencing

Herbarium samples were obtained from Moscow University Herbarium (Seregin, 2020). Extraction of DNA from herbarium samples of one *Arabidopsis suecica* accession and five *A. arenosa* individuals was carried out under a PCR workstation equipped with UV sterilization lights. The workstation, tubes, and equipment were UV‐sterilized before beginning the extraction. Herbarium samples were weighed and photographed. The material was then crushed using a sterile plastic pestle in a 2‐ml tube and then weighed again. Phenacylthiazolium Bromide (PTB) buffer was added, and the material was kept at 37°C overnight on continuous rotation. We extracted the DNA following the protocol by Gutaker *et al*. (2017). Libraries were sequenced in 50‐bp paired‐end mode on Illumina HiSeq 2000 Analyzers at the Vienna BioCenter Core Facilities (VBCF).

### Read mapping and SNP calling

For *A. thaliana* accessions, 966 accessions of *A. thaliana* were used from the 1001 genomes dataset (1001 Genomes Consortium, 2016). These accessions had a read length > 60 bp and > 8× coverage. In addition to the aforementioned herbarium samples, we used 15 previously published *A. suecica* accessions (Novikova *et al*., 2017). For *A. arenosa*, we used 158 previously published individuals (Monnahan *et al*., 2019; Bohutínská *et al*., 2023a) in addition to the five herbarium *A. arenosa* individuals sequenced here, giving a total of 163 *A. arenosa* individuals. The *A. arenosa* individuals used had a minimum of 5× coverage. A complete list of the *A. thaliana* and *A. suecica* accessions and the *A. arenosa* individuals used in this study is available as Supporting Information Dataset S1.

We mapped Illumina paired‐end short reads for the 16 *A. suecica* accessions, 970 *A. thaliana* accessions, and 163 *A. arenosa* individuals to a chromosome‐level genome assembly of *A. suecica* (Burns *et al*., 2021) using the BWA–MEM algorithm with an increased penalty for unpaired reads set to 15 (Li & Durbin, 2009), v.0.7.17. The sub‐genomes of the *A. suecica* accessions were treated separately after partitioning the mapped short reads. The *A. thaliana* accessions and *A. arenosa* individuals were mapped to the *A. thaliana* and *A. arenosa* sub‐genomes, respectively. PCR duplicates were removed using the rmdup function from samtools v.1.10 (Li *et al*., 2009). Single nucleotide polymorphisms (SNPs) were called using Genome Analysis Toolkit (GATK) v.3.8 (McKenna *et al*., 2010), using the HaplotypeCaller function. Generated gVCFs were combined into a single merged VCF using the combineGVCFs and GenotypeGVCFs functions.

### Variant filtration

We removed multi‐allelic sites and indels from our analysis and focused on biallelic SNPs. Biallelic SNPs were filtered to remove repetitive regions in the *A. suecica* sub‐genomes (TEs sequences, centromere repeats, simple repeats, and rDNA repeats). SNPs and nonvariant sites were removed if the site was < −0.5× log median coverage or > 0.5× log median coverage. The coverage thresholds were chosen to exclude sites with abnormally low or high coverage. Low coverage may indicate poor read mapping or insufficient sequencing depth, while high coverage can signal repetitive elements or cryptic duplications, or potential homeologous exchange events, all of which can lead to erroneous variant calls.

Heterozygous SNPs in *A. thaliana* have previously been shown to often result from segregating gene duplications rather than being residual heterozygosity from outcrossing (Jaegle *et al*., 2023). To avoid SNP‐calling errors from segregating gene duplications in our VCF, we removed heterozygous SNPs that were present in *A. thaliana* and *A. suecica* at a population frequency > 5%, since both are highly selfing species. As *A. arenosa* is an obligate outcrossing species, to avoid segregating duplicates, we removed heterozygous SNPs that were heterozygous in > 90% of the individuals in the *A. arenosa* populations we examined. We allowed for 10% missing sites in the *A. arenosa* VCF. We allowed for 15% missing sites in the *A. thaliana* VCF.

The final VCFs contained 9664 305 biallelic SNPs for the *A. arenosa* sub‐genome, and 6125 591 biallelic SNPs for the *A. thaliana* sub‐genome.

### Determining the ploidy level of *A. arenosa* individuals

The cytotype of the published *A. arenosa* individuals' ploidy was previously confirmed using FACs (Monnahan *et al*., 2019; Bohutínská *et al*., 2023). nQuire (Weiß *et al*., 2018) was run on raw fastq files of the five herbarium *A. arenosa* individuals, using the ‘denoised’ function and the ‘lrdmodel’. Ploidy of the sample was determined by taking the ploidy that gave the maximum log‐likelihood and the lowest delta log‐likelihood. Ploidy of the herbarium *A. arenosa* individuals was further confirmed by counting the number of different *S*‐alleles for each individual, with tetraploids having up to four and diploids up to two different *S*‐alleles. The S‐alleles were identified as described in Kolesnikova *et al*. (2023) using short‐read data and the NGSGenotyp pipeline (Genete *et al*., 2020) with known *Arabidopsis* SRK sequences as a database.

### Nucleotide divergence analysis

Pairwise nucleotide diversity (π) was calculated using custom scripts (see Code availability) in Python. In order to carry out the network based on Nei's D, we read the VCF into R using the package vcfr (Knaus & Grünwald, 2017) and calculated Nei's D using StAMPP (Pembleton *et al*., 2013). To visualize the network, we used the R package tangle (Schliep *et al*., 2021) and ggtree (Yu, 2020). Geographic maps were made using the ggspatial package in R (Dunnington, 2025).

### Niche modeling of *A. suecica*

We modeled the niche of *A. suecica* using the current occurrence records and the selected Bioclim variables to project it onto the climate at Last Glacial Maximum (LGM). We used the workflow described in the modler package in R (Sánchez‐Tapia *et al*., 2020) for the entire modeling (setup, evaluation, and projection).

We obtained occurrence data of *A. suecica* from the GBIF database (gbif.org). There are a total of 8451 records available, of which we considered records only from preserved specimens having georeferencing data (1772 records). These data were supplemented with the published records (Novikova *et al*., 2017). After cleaning the data for duplicates and occurrences in the same pixel, 483 clean data points were obtained. Then, species thinning was carried out using the geo_filt function embedded in ModleR. Finally, 197 data points remained after applying the geographic filter of 0.5 km. These occurrence points were considered for further steps. We created 10 000 background points using the setup_sdmdata function in the modler package.

We obtained 19 bioclim variables from Worldclim climate data (Fick & Hijmans, 2017) at a resolution of 30 arc seconds. We extracted the data for our occurrence points using the R package raster (Hijmans, 2018). Multicollinearity was checked for the bioclim variables in order to enhance the model consistency and remove the impact of highly correlated variables. Out of the 19 variables, eight uncorrelated variables (bio3, bio4, bio8, bio9, bio10, bio14, bio15) were then used for further analysis.

We used maxEnt to fit our model, which uses the occurrence data and the corresponding climatic data to predict the probability of distribution of the target species. Twenty‐five models were created by partitioning the data into five cross‐validation partition types and five runs per partition. We evaluated the performance of these models using the true skill statistic (TSS), which is based on the confusion matrix (Allouche *et al*., 2006). The TSS scores span from 0 to 1, where 0 signifies a model performing no better than chance, and 1 denotes a flawless alignment with the observed data. We had set a TSS value > 0.7 to prepare the final model. Since all our models showed TSS values > 0.9, we considered all 25 models.

The final model was projected onto the past climate (LGM). Further, occurrence points of *A. thaliana* and *A. arenosa* were overlaid on the projection to understand the origin and distribution of *A. suecica* environmentally and spatially.

### Selection scans

To examine polymorphism patterns in genes, we first ran SnpEff for annotation, and we used a modified version of the McDonald–Kreitman (MK) test in order to find genes showing signatures of selection in *A. suecica*. We used a modified version of the MK test, as *A. suecica* is evolutionarily young (*c*. 16 Kya), meaning if we relied on ratios of divergence to polymorphism in the MK test, we would often have division by zero. Therefore, we focused instead on genes where nonsynonymous divergence (Dn) exceeded synonymous divergence (Ds), nonsynonymous polymorphisms (Pn) were fewer than synonymous polymorphisms (Ps), and where Pn was zero in *A. suecica*.

### GO enrichment

We used the R package topgo (Adrian Alexa, 2017) to perform our Gene Ontology (GO) enrichment analysis, using the ‘weight01’ algorithm to account for the hierarchical structure in GO terms. We kept GO terms with a Fisher test *P*‐value <0.05. We used the gene annotations of *A. thaliana* one‐to‐one orthologs of *A. suecica* genes. Gene annotations for *A. thaliana* were obtained using the R package biomaRt from Ensembl ‘biomaRt::useMart(biomart = ‘plants_mart’, dataset = ‘athaliana_eg_gene’, host = ‘plants.ensembl.org’).

### SNP annotation, polarization, and homeolog analysis

We annotated SNPs using SNPeff (v.5.2). We used 200 of the genetically closest *A. thaliana* accessions to polarize annotated SNPs on the *A. arenosa* sub‐genome. We used 29 individuals of the closest diploid *A. arenosa* to polarize annotated SNPs on the *A. thaliana* sub‐genome. This resulted in 3500 145 annotated and polarized SNPs for the *A. arenosa* sub‐genome and 1205 789 for the *A. thaliana* sub‐genome. 11 812 and 11 983 SNPs, on the *A. arenosa* and *A. thaliana* sub‐genomes, respectively, were annotated as predicted LoF polymorphisms that were later used in our homeolog analysis. Predicted LoF polymorphisms were selected by taking SNPs annotated by SNPeff with a high effect. We excluded SNPs with heterozygosity frequencies exceeding 1% in both *A. thaliana* and *A. suecica* to minimize the risk of bias in the LoF mutation analysis, which could be introduced by segregating gene duplicates leading to false signals of heterozygosity. We filtered for homeolog pairs where in *A. suecica* at least one homeolog in the pair carried a LoF mutation, amounting to 414 genes. In instances where a homeolog carried more than one LoF mutation, we selected the LoF mutation that was at a higher population frequency.

### Permutation analysis

To assess the negative correlation of LoF mutations between the *A. thaliana* and *A. arenosa* sub‐genomes in *A. suecica*, we conducted two complementary permutation analyses. First, we compared the observed number of homeologous gene pairs with LoF mutations in both sub‐genomes to expectations from random pairings. Specifically, we focused on 414 homeolog pairs in which at least one gene copy carried a LoF polymorphism in *A. suecica*. These 414 homeolog pairs represent the subset of the genome where LoF variation exists and where compensatory dynamics can be meaningfully assessed. We did not include all homeologs in the permutation, as the rarity of LoF mutations would heavily skew the null distribution toward zero co‐occurrence, limiting the sensitivity of the test. From this filtered set, we generated 1000 permutations by randomly re‐pairing homeologs and compared the observed number of dual LoF pairs to the resulting distribution.

Second, we performed a permutation analysis using simulated *in‐silico A. suecica* genomes. We generated 1000 *in‐silico A. suecica* by randomly combining genomes from 200 *A. thaliana* and 29 diploid *A. arenosa* individuals that are genetically closest to natural *A. suecica*, sampling with replacement to account for all possible combinations. For each simulated genome, we assessed dual LoF occurrence in the same set of 414 homeolog pairs as in the natural *A. suecica* dataset. The observed number of homeolog pairs with LoF mutations in both sub‐genomes was then compared with the distribution derived from the *in‐silico* genomes, providing an additional null expectation under neutral inheritance.

For both tests, statistical significance was determined by comparing the observed number of dual LoF pairs in natural *A. suecica* to the empirical null distribution generated by the permutation.

### Purifying selection analysis

To evaluate evidence of purifying selection acting on intact homeologs relative to their LoF carrying counterpart, we quantified pairwise nucleotide diversity (π) using coding sequences from *A. suecica*. We further calculated the ratio of nonsynonymous (Pn) to synonymous (Ps) polymorphisms to assess selective constraint. Given the severe population bottleneck in *A. suecica*, a pseudocount of 0.1 was added to both Pn and Ps to avoid division by zero. Statistical significance was determined using a one‐sided paired *t*‐test, testing the hypothesis that intact homeologs have reduced π and Pn/Ps compared with their LoF pairs.

### Analysis of cell cycle genes in the *A. suecica A. arenosa* sub‐genome

Biallelic SNPs from coding sequence (CDS) regions of the genes were used to generate SNP‐frequency heatmaps. *A. arenosa* assemblies from (Bohutínská *et al*., 2023a) were scaffolded with RagTag (Alonge *et al*., 2022) on the *A. lyrata* NT1 genome assembly (Kolesnikova *et al*., 2023) and annotated with Helixer (Stiehler *et al*., 2021). *A. thaliana* protein sequences of target genes were used for protein BLAST (Camacho *et al*., 2009) against annotated proteins extracted with AGAT (Dainat *et al*., 2024). Genes identified by BLAST were aligned with the L‐INS‐i algorithm of MAFFT (Katoh *et al*., 2005), and trees were constructed with IQTree (Nguyen *et al*., 2015; Hoang *et al*., 2018) with the fast bootstrap option equals 1000. Protein alignments were visualized using snipit (O'Toole *et al*., 2024).

### Single‐ and multi‐copy gene analysis

Single‐copy and multi‐copy genes were obtained from Li *et al*. (2016), and hypergeometric tests were performed to test for enrichment.

### Expression analysis

To test whether transcription is biased toward the intact member of a homeologous pair, we mined log₂‐fold‐change (log₂FC) estimates for rosette tissue from Burns *et al*. (2021) which include 15 natural *A. suecica* accessions and two synthetic lines. We first averaged log_2_FC values within each group (natural vs synthetic). We then restricted the analysis to homeolog pairs in which the LoF allele segregates at high frequency in natural *A. suecica* (≥60%). This filter retained 64 intact homeologs, 16 on the *A. arenosa*‐derived sub‐genome and 48 on the *A. thaliana*‐derived sub‐genome, out of the 414 total pairs.

### STRING network analysis

To investigate protein–protein association networks among genes identified in our selection scan of the *A. arenosa* sub‐genome of *A. suecica*, we used the STRING database (Szklarczyk *et al*., 2023). To identify gene groups with shared function and protein–protein associations, we applied Markov cluster algorithm (MCL) clustering, using an inflation parameter of 2, resulting in 102 discrete clusters. Four of the 102 clusters were investigated further due to their association with important polyploid traits.

## Results

### Genetic origins of *A. suecica*

To reconstruct the evolutionary history of allotetraploid *A. suecica*, we first aimed to identify the closest parental accessions, using available data from the parental species, *A. thaliana* and *A. arenosa*, covering their vast geographic range. We mapped 966 *A. thaliana* accessions (1001 Genomes Consortium, 2016) and 163 *A. arenosa* individuals (93 tetraploids and 70 diploids), representing spatially close lineages of both diploid and autotetraploid cytotypes (Novikova *et al*., 2016; Monnahan *et al*., 2019; Bohutínská *et al*., 2024), to the *A. thaliana* and *A. arenosa* sub‐genomes of *A. suecica* (Burns *et al*., 2021). Genomic positions were conservatively filtered to ensure reliable variant calling (see the Materials and Methods section). In summary, callable sites included *c*. 62% (73 813 230) of the *A. thaliana* sub‐genome and *c*. 34% (47 950 105) of the *A. arenosa* sub‐genome, with 6 125 591 biallelic SNPs in *A. thaliana* and 9 664 305 in *A. arenosa*. Throughout this study, results generated for the sub‐genomes of *A. suecica* are compared with corresponding data from the parental species, *A. thaliana* and *A. arenosa*.

**Fig. 1:**
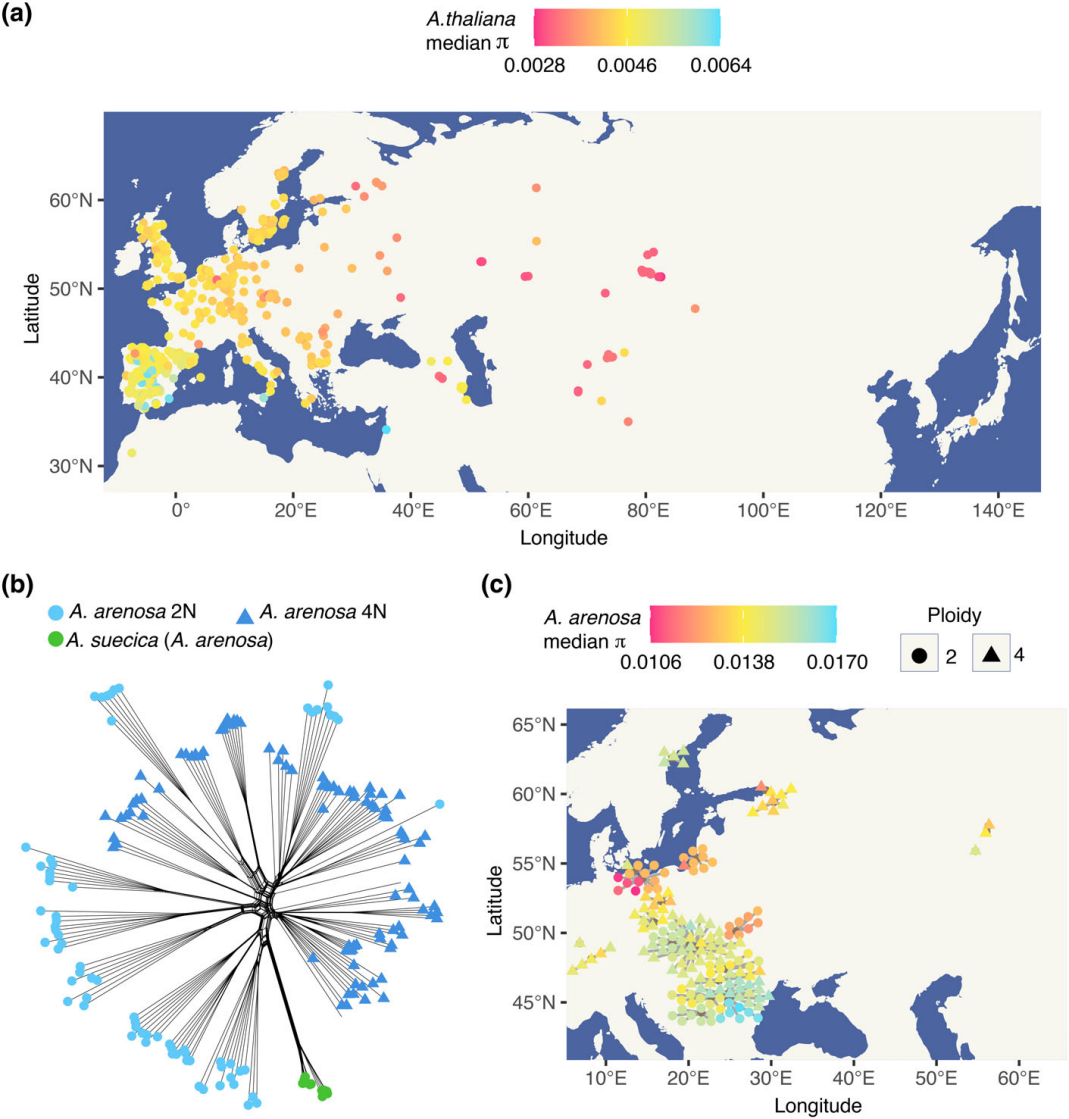
The genetic and geographic origin of *Arabidopsis suecica*. (a) Median nucleotide diversity (π) of 970 *Arabidopsis thaliana* accessions to 16 *A. suecica* accessions shows Central Eurasian *A. thaliana* are genetically closest. (b) A genetic distance network (Nei's D) shows that *A. suecica* (green circles) is genetically closer to diploid *Arabidopsis arenosa* (light blue circles) than autotetraploid *A. arenosa* (dark blue triangles). (c) Median nucleotide diversity (π) of *A. arenosa* individuals to 16 *A. suecica* accessions indicates that Southern Baltic *A. arenosa* diploids (red circles) are the genetically closest. Locations are represented by gray lines connecting *A. arenosa* individuals to their geographic origins.

Previously, it was shown that the closest *A. thaliana* accessions to *A. suecica* are found in Central Eurasia (Novikova *et al*., 2017). Here, eliminating the possibility of a reference bias artifact, we calculated median pairwise diversity (π) for each *A. thaliana* accession to our 16 *A. suecica* natural accessions and confirmed that result (Fig. 1a). To identify the *A. arenosa* individuals most closely related to *A. suecica* and to determine their cytotype (diploid or tetraploid), we expanded the previously released *A. arenosa* dataset (Monnahan *et al*., 2019; Bohutínská *et al*., 2024). We sequenced five additional autotetraploid *A. arenosa* individuals sampled from herbarium specimens collected in the southern Urals, representing the easternmost populations and those geographically closest to the parental *A. thaliana* accessions. The southern Ural *A. arenosa* individuals were identified as tetraploids, using the software nQuire (Weiß *et al*., 2018) and counting the number of different *S*‐alleles (see Materials and Methods section, similar to ploidy determination in *A. lyrata* [Scott *et al*., 2025]), while the ploidy of the remaining *A. arenosa* samples was previously determined using flow cytometry (Monnahan *et al*., 2019; Bohutínská *et al*., 2024).

We conducted a network analysis based on genetic distances (Nei's D (Nei, 1972)) that are suitable for mixed‐ploidy datasets (Pembleton *et al*., 2013) (Fig. 1b). We also calculated the median pairwise nucleotide diversity (π) for each *A. arenosa* individual against our 16 *A. suecica* natural accessions and placed it on a geographical map (Fig. 1c). Our results indicate that diploid *A. arenosa* from the southern Baltic Sea coast (Kolář *et al*., 2016) is genetically the closest to *A. suecica* and certainly closer than any autotetraploid *A. arenosa*, including both the current sympatric tetraploid Ruderal lineage growing in Fennoscandia (Monnahan *et al*., 2019) and tetraploid populations from the southern Urals, that is, the geographic region closest to the putative ancestral *A. thaliana* lineage (Fig. 1a). A full list of *A. thaliana* and *A. arenosa* accessions and their π values to *A. suecica* is available in Dataset S1. The vast majority of the SNPs (> 99%) in *A. suecica* are shared with *A. arenosa* and *A. thaliana*, indicating that polymorphisms in *A. suecica* were largely inherited from standing variation in multiple, genetically distinct parental individuals, rather than arising from *de novo* mutation, in agreement with previous reports (Novikova *et al*., 2017) (Fig. S1).

Together, our results suggest a diploid rather than a tetraploid origin for the *A. arenosa* sub‐genome within *A. suecica*. This is in agreement with data showing that meiotic genes on the *A. arenosa* sub‐genome of *A. suecic*a carry sequence polymorphisms characteristic of diploid *A. arenosa*, and not the polymorphisms found in autotetraploid *A. arenosa* (Nibau *et al*., 2022). The diploid origin supports the original hypothesis by Hylander in 1957 (Hylander, 1957) that *A. suecica* originated from hybridization between diploid *A. thaliana* and diploid *A. arenosa*, with autotetraploid *A. arenosa* later spreading across Fennoscandia, while the diploids remain restricted to southeastern Europe and isolated Baltic areas (Kolář *et al*., 2016). The lack of geographical overlap between the current distribution of the closest *A. thaliana* and *A. arenosa* progenitors raises the question of where the hybridization could have occurred? To resolve this question we performed ecological niche modeling in the context of the estimated time of origin of *A. suecica*, that is, toward the end of the Last Glacial Maximum (Novikova *et al*., 2017), and we suggest that hybridization between *A. thaliana* and *A. arenosa* likely happened close to the border of the Fennoscandia ice sheet (see Notes S1; Fig. S2).

### Genomic adaptation to allopolyploidy in *A. suecica*

Given that the genetically closest parents of *A. suecica* were diploids, *A. suecica* could not inherit any of the potentially pre‐adaptive meiotic alleles from autotetraploid *A. arenosa*. Previously, we reported that meiosis‐related genes on the *A. thaliana* sub‐genome of *A. suecica* are upregulated compared with parental transcriptional levels (Burns *et al*., 2021). However, in the same study, we did not observe significant upregulation of meiosis‐related genes on the *A. arenosa* sub‐genome. Notably, the latter transcriptional comparison was made against tetraploid *A. arenosa*, the previously hypothesized parent of *A. suecica*, whereas we now establish here that diploid *A. arenosa* is closer. Therefore, to uncover how *A. suecica* adapted to allopolyploidy from diploid ancestry, we searched for signatures of selection on genes, performing the MK test between *A. suecica* and the identified closest progenitors (see the Materials and Methods section). While this approach highlights strong candidates for selection on coding sequences, we note that it will miss selection acting on regulatory elements. Homeologous recombination events could also inflate divergence estimates at some loci; however, we applied coverage‐based filters during SNP calling to avoid such events (see the Materials and Methods section). Additionally, homeologous exchange (HE) events are rare in natural *A. suecica*, with only two homoeologous exchange junctions reported in Burns *et al*. (2021) and nine in Zhang *et al*. (2020), in a population of *A. suecica*. HE events are also distally biased toward subtelomeric regions (Zhang *et al*., 2020; Nibau *et al*., 2024). By contrast, our selection‐scan genes do not show such a bias (see Fig. S2), indicating that HEs are unlikely to drive our selection‐scan results. Additionally, gene conversion rates in *A. thaliana* are similarly low, estimated at *c*. 3.6 × 10^−6^ per site per meiosis (Wijnker *et al*., 2013).

Using the MK test, we identified signatures of selection on 35 genes in *A. thaliana* and 585 genes in the *A. arenosa* sub‐genomes of *A. suecica* (Dataset S2). Due to the low number of founders contributing to the origin of *A. suecica* (Novikova *et al*., 2017), regions of the genome are depleted of genetic diversity; however, we find that many of the genes under selection are located outside of these low‐diversity regions (Fig. S3) and show GO enrichment, supporting their biological relevance. Enriched categories include meiotic cell cycle (GO:0061780) and mitotic cohesin loading (GO:0051321); however, some are driven by individual genes (Fig. S4; Dataset S2), for example, SCC2 alone drives the mitotic cohesin loading term, and so this single‐gene‐driven GO term limits a broader interpretation. We note that genes under selection on the *A. arenosa* sub‐genome do not overlap with the previously described BYS QTL locus for meiotic stability in *A. suecica* (Henry *et al*., 2014).

**Fig. 2:**
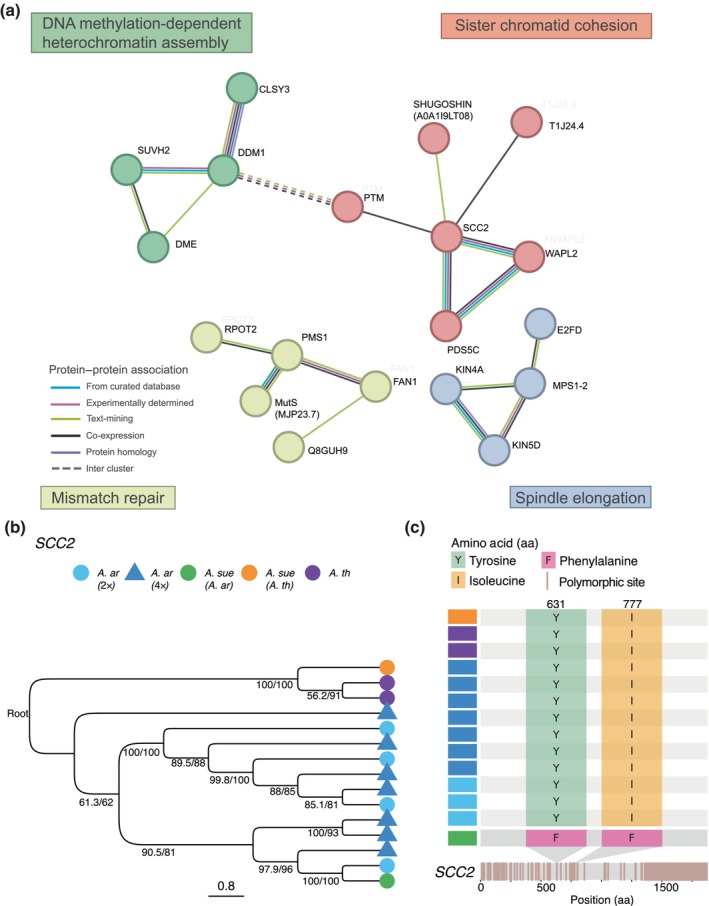
Genomic adaptation to allopolyploidy in *Arabidopsis suecica*. (a) STRING protein–protein association network showing four functional clusters among 19 genes under positive selection on the *Arabidopsis arenosa* sub‐genome of *A. suecica*, as identified by McDonald–Kreitman tests. Colored clusters represent functional clusters, including DNA methylation‐dependent heterochromatin assembly (green), sister chromatid cohesion (red), mismatch repair (yellow), and spindle elongation (blue). Solid lines represent intra‐cluster associations; dotted lines indicate inter‐cluster associations. (b) Phylogenetic tree from protein alignment of SISTER CHROMATID COHESION2 (SCC2) showing that the *A. arenosa* homeolog in *A. suecica* clusters with diploid *A. arenosa*. Branch length is not drawn to scale to improve the analysis of the tree topology. Both the translated genomic and coding sequence (CDS) sequence of the *SCC2* from *Arabidopsis thaliana*, in addition to the *A. suecica*, *A. thaliana* homeolog were used to root the tree. Numbers on the nodes represent branch support values from IQTree analyses. SH‐aLRT (Shimodaira‐Hasegawa liklihood ratio test) support on the left and ultrafast bootstrap (UFBoot) on the right.  (c) Fixed amino acid differences in the *SCC2* between the *A. suecica*, *A. arenosa* sub‐genome and diploid *A. arenosa*. Tetraploid *A. arenosa* and *A. thaliana* share the same amino acid as diploid *A. arenosa*; the difference is unique to *A. suecica*.

To better explore how positively selected genes might functionally interact, we used the STRING database (Szklarczyk *et al*., 2023) to construct a protein–protein association network (Fig. 2a; see the Materials and Methods section). Among the 585 genes under selection in the *A. arenosa* sub‐genome of *A. suecica*, 19 formed a connected subnetwork in STRING. These genes clustered into four distinct functional clusters identified using Markov Clustering (MCL), corresponding to sister chromatid cohesion, DNA methylation‐dependent heterochromatin assembly, mismatch repair, and spindle elongation. The sister chromatid cohesion cluster contains six interacting proteins, including *SCC2*, *WAPL2*, and *SHUGOSHIN*. The DNA methylation cluster includes four genes, among them *SUVH2* and *DDM1*. The mismatch repair cluster (5 genes) contains *MutS* and *PMS1*, and the spindle elongation cluster (4 genes) includes *MPS1‐2*. The four functional clusters are supported by multiple protein–protein association evidence types, including experimentally determined, co‐expression, protein homology, and curated databases (Fig. 2a legend). By contrast, the 35 selected genes from the *A. thaliana* sub‐genome did not form a connected network or enriched functional clusters.

Among the four clusters, the sister chromatid cohesion cluster stood out because of the importance of cohesion and the cohesin complex in meiosis (Wang *et al*., 2020; Storchová *et al*., 2006). *SCC2* (SISTER CHROMATID COHESION2), which encodes the cohesin loader adherin, a protein essential for cohesin loading onto chromosomes (Wang *et al*., 2020), emerged as a particularly compelling candidate because *SCC2* has also previously been implicated in adaptation in autotetraploid *Arabidopsis arenosa* (Yant *et al*., 2013). To investigate whether SCC2 represents the same haplotype as that of tetraploid *A. arenosa* or instead represents *de novo* evolution within *A. suecica* following allopolyploidization, we performed a phylogenetic analysis, which showed that *A. suecica* clusters consistently with diploid *A. arenosa* (Fig. 2b) and does not show evidence of haplotype sharing with autotetraploid *A. arenosa*. Furthermore, *A. suecica* has private polymorphisms on SCC2 (*A. arenosa* sub‐genome), which are not present in either diploid or tetraploid *A. arenosa* or *A. thaliana* (Fig. 2c), suggesting *de novo* evolution.

### Preservation of gene function via homeolog compensation in *A. suecica*

Polyploid organisms have a substantial amount of genetic redundancy, which can lead to relaxed purifying selection. This pattern of relaxed selection has been observed in autopolyploid *A. arenosa* (Baduel *et al*., 2019; Vlček *et al*., 2025) and allopolyploid *A. kamchatica* (Paape *et al*., 2018). We investigated whether *A. suecica*, the youngest polyploid in the *Arabidopsis* genus, shows a similar signal of relaxed purifying selection and asked how patterns of LoF mutations are distributed across its sub‐genomes.

**Fig. 3:**
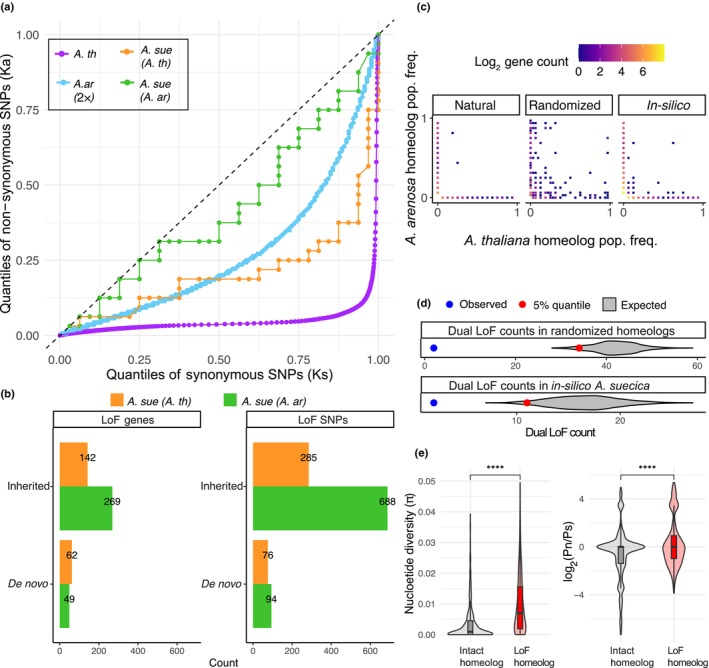
Relaxed purifying selection and homeolog compensation in *Arabidopsis suecica*. (a) Quantile‐quantile (QQ) plot of synonymous (Ks) and nonsynonymous (Ka) single‐nucleotide polymorphism (SNP) frequencies for *A. suecica* and its parent species (*Arabidopsis thaliana* and diploid *Arabidopsis arenosa*). The dotted line indicates neutral evolution. All species exhibit purifying selection, with the *A. suecica* sub‐genomes showing relaxed purifying selection relative to the parents. (b) Bar plots showing the number of genes and total counts of loss‐of‐function (LoF) SNPs in *A. suecica*, the majority of which are inherited from the parent species. (c) Joint frequency spectra of homeolog pairs with at least one LoF SNP, displaying population frequencies for *A. thaliana* and *A. arenosa* homeologs on the x‐axis and y‐axis, respectively. From left to right: observed spectrum in natural *A. suecica*, example from permutations of randomized homeologs, and *in silico A. suecica* controls (random combinations of *A. thaliana* and *A. arenosa* individuals). The number of homeologs analyzed is the same (414 homeologs), but the distribution of LoF mutations is different between the three panels. (d) Comparison of natural *A. suecica* with 1000 randomized and *in silico* controls, showing that *A. suecica* (blue dot) has fewer dual LoF SNPs in homeolog pairs than expected by chance (gray violin plot), a difference that is statistically significant (red dot denotes *P*‐value = 0.05). (e) Evidence of purifying selection acting on the intact homeolog, which shows significantly lower nucleotide diversity (π) and a lower ratio of nonsynonymous (Pn) to synonymous polymorphisms (Ps) than the LoF homeolog, within *A. suecica*. Statistical significance is indicated by asterisks (one‐sided *t*‐test). Whiskers extend to 1.5× the interquartile range (IQR), and the violin outline gives the density of the data distribution. The box within the violin plot gives the IQR range and the horizontal black line inside the boxplot gives the median value.

Analyzing over 3.5 million SNPs in the *A. arenosa* sub‐genome and 1.2 million SNPs in the *A. thaliana* sub‐genome, we found that both sub‐genomes in *A. suecica* are evolving under purifying selection; however, purifying selection appears relaxed in *A. suecica* compared with the parental species (Fig. 3a). This genome‐wide signal suggests a reduced selective constraint following polyploidization, consistent with the buffering effects of homeolog redundancy.

A reduced selective constraint following polyploidization enables mutational robustness between the sub‐genomes, since a LoF mutation in one sub‐genome can be compensated by its homeolog on the alternate sub‐genome. To investigate the compensatory relationship between homeologs, we identified a total of 414 homeologous gene pairs where at least one copy carries a segregating LoF mutation in *A. suecica*. To understand the nature of LoF mutations between homeologs, we split them into inherited (standing variation) and *de novo* (occurring post‐allopolyploidization) LoF mutations in *A. suecica*. While a slight excess of LoFs was found in *A. arenosa*‐derived homeologs (269 vs 142), the majority of these mutations are also present in the parental genomes and thus appear to be inherited rather than arising *de novo* (Fig. 3b). Focusing only on *de novo* LoF mutations unique to *A. suecica*, the distribution appears more balanced (49 in *A. arenosa*, 62 in *A. thaliana*). To investigate if these LoF homeologs overlap with genes identified in our selection scans for genomic adaptation to allopolyploidy in *A. suecic*a (as mentioned in the previous section), we compared the genes involved. However, we find minimal overlap between the gene lists (Fig. S5), indicating that a LoF occurring on one homeolog is not driving the signals of positive selection we observed in the previous section.

We next asked if LoF mutations (both inherited and *de novo*) observed in 414 homeologous gene pairs are distributed randomly or whether having a LoF mutation in one homeolog would interfere with the occurrence of LoF in the other homeolog. The latter would be explained by purifying selection acting to preserve at least one functional copy in *A. suecica*. To test this, we compared the observed joint frequency spectrum of LoF mutations between the *A. suecica* sub‐genomes to (1) spectra resulting from reshuffling of homeolog pairings and (2) the *A. suecica* sub‐genomes to spectra of *in silico* synthetic *A. suecica* genomes generated by combining natural diploid *A. arenosa* and *A. thaliana* individuals (Fig. 3c,d). In both comparisons, we found that dual LoF mutations are significantly less frequent in natural *A. suecica* (Fig. 3c,d; *P* < 0.05). The depletion of dual LoFs supports a model of compensatory homeolog retention, in which functional redundancy between homeologs allows relaxed selection at the level of individual genes, while purifying selection operates at the pair level to prevent the complete loss of gene function. To further test for evidence of purifying selection acting on the remaining intact homeolog, we compared pairwise nucleotide diversity (π) and the ratio of nonsynonymous to synonymous polymorphisms (Pn/Ps) between our 414 homeolog pairs. We found that intact homeologs exhibited significantly lower π and Pn/Ps values than the paired homeolog carrying a LoF mutation, consistent with purifying selection acting on the intact copy (*P* = 2.14 × 10^−26^ for π; *P* = 5.15 × 10^−10^ for Pn/Ps; one‐sided *t*‐tests Fig. 3e). Supporting this further, we also observed sub‐genome‐biased expression favoring the intact copy, a pattern absent in synthetic *A. suecica*, suggesting it is an evolved rather than inherited expression change (Fig. S6). We observed no consistent bias in retention from either sub‐genome, suggesting that functional constraint, rather than sub‐genome dominance, is the primary force shaping homeolog retention in *A. suecica*.

Genes that consistently revert to a single‐copy state following WGD are thought to be under strong evolutionary constraint, likely because they function optimally at single‐copy dosage and are sensitive to the effects of gene duplication (Edger & Pires, 2009; Makino & McLysaght, 2010). To test whether such dosage constraints influence homeolog retention in *A. suecica*, we asked whether genes previously identified as evolving toward single‐copy status across angiosperms (Li *et al*., 2016) are overrepresented among homeologs carrying LoF mutations. We also tested whether LoF mutations in these single‐copy genes occur at higher frequencies in *A. suecica* populations compared with those in genes typically retained in multiple copies. Using the published gene set from Li *et al*. (2016), we observed no significant enrichment of single‐copy genes among homeologs carrying a LoF mutation nor do we see that LoF mutations within single‐copy genes exist at higher population frequencies than LoF mutations within multi‐copy genes in *A. suecica* (Fig. S7). These results suggest that constraint on single‐copy gene retention is not a major factor shaping homeolog loss of function in *A. suecica*, at least at this stage of genome evolution.

In the three *A. suecica* homeolog gene pairs where we do observe segregating dual LoF mutations, we tested whether functionally redundant copies from older WGDs might compensate for complete gene function loss. Using ancient homeolog pairs from *A. thaliana* (Guo *et al*., 2013), we found no overlap with our dual LoF genes, suggesting these losses are not buffered by retained duplicates from older WGDs. The low frequency of dual LoF mutations (3 out of 414 gene pairs) supports the action of strong purifying selection in the population against the complete loss of gene function.

## Discussion

### A diploid origin for *A. suecica*

*Arabidopsis suecica* is a relatively young allopolyploid species that originated from the hybridization of *A. thaliana* and *A. arenosa*. Using population‐level DNA sequencing for all three species, we revisited the genetic origins of *A. suecica* to understand the nature of WGD leading to the formation of the allopolyploid species and whether the ancestry shaped adaptation to allopolyploidy.

Leveraging the recent genome assembly of *A. suecica* (Burns *et al*., 2021), we minimized reference bias and identified the genetically closest individuals of both progenitor species. Consistent with earlier reports (Novikova *et al*., 2017), we confirm that the *A. thaliana* parent was diploid. However, we now find that the *A. arenosa* lineage closest to *A. suecica* is also diploid, not tetraploid. Together, this supports a model in which *A. suecica* originated via somatic genome doubling in a diploid hybrid background, rather than through fusion of unreduced gametes. A similar pattern is also seen in *A. kamchatica*, another allopolyploid *Arabidopsis* species, which also derives from diploid progenitors (Kolesnikova *et al*., 2023; Scott *et al*., 2025). These findings suggest that somatic doubling of diploid hybrids may be a general mechanism for allopolyploid origin in *Arabidopsis*.

### Evolution of meiosis and genome stability in *A. suecica*

Our STRING network revealed four functional clusters: sister chromatid cohesion, DNA methylation, spindle elongation, and mismatch repair, which together suggest genomic adaptation to the challenges of recombination and chromosome segregation in allopolyploid meiosis.

The sister chromatid cohesion cluster includes *SCC2* and *WAPL2*, which are central regulators of cohesin dynamics. *SCC2* encodes the conserved cohesin loader required for the deposition of cohesin complexes along chromosomes (Wang *et al*., 2020). In *A. thaliana*, SCC2 localizes to meiotic chromosome axes and is essential for axis formation, synapsis, and homolog pairing (Wang *et al*., 2020). *WAPL2*, conversely, promotes cohesin removal, allowing for the timely release of cohesion during meiotic prophase I (Srinivasan *et al*., 2019; Wang *et al*., 2020) and in *A. thaliana*, the loss of *WAPL* leads to unresolved bivalents and uneven chromosome segregation (De *et al*., 2014). *Shugoshin* has an important role in protecting cohesion at centromeres at anaphase I (Watanabe & Kitajima, 2005). Together, these genes support the functional importance of cohesion and the cohesin complex in polyploid meiosis. We note that SCC2 shows independent signatures of selection in both *A. arenosa* and *A. suecica*, suggesting its general importance in relation to polyploid genome stabilization.

The DNA methylation cluster contains *SUVH2* and *DDM1*. *SUVH2* has been previously shown to play an important role in suppressing the transcription of genes that are associated with meiotic double‐strand breaks (DSBs) (Wang *et al*., 2022), while a deficiency in *DDM1* has been shown to lead to an increase in meiotic crossovers in *A. thaliana* (Melamed‐Bessudo & Levy, 2012). Their selection in *A. suecica* suggests the importance of chromatin in allopolyploids, possibly by restricting DSBs and recombination to appropriate genomic regions.

The mismatch repair cluster contains the key genes *MutS* and *PMS1*. In allohexaploid wheat, the *Ph2* locus encodes a *MutS* homolog *MSH7‐3D*, which has been shown to suppress homeologous recombination by destabilizing homeologous interactions (Serra *et al*., 2021). *MSH7‐3D* is thought to influence recombination partner choice and favor homologous over homeologous chromosomes by destabilizing recombination intermediates that contain mismatches (Serra *et al*., 2021). In yeast, *PMS1* has been shown to reduce recombination between homeolog chromosomes in meiosis (Chambers *et al*., 2023) and loss of MMR activity in *A. thaliana* has been shown to increase homeologous recombination nine‐fold (L. Li *et al*., 2006).

The spindle elongation cluster, which includes the cell cycle kinase *MPS1‐2*, is essential for spindle pole body duplication and the spindle assembly checkpoint (Jiang *et al*., 2009). Polyploid yeast have been shown to be critically dependent on *MPS1‐2* for successful chromosome segregation (Meyer *et al*., 2023) suggesting a conserved role in supporting meiotic stability in polyploid genomes.

Taken together, these four clusters suggest a coordinated mechanism by which *A. suecica* has adapted its meiotic machinery to maintain genome stability following allopolyploidization. First, cohesion regulators and cohesin‐associated proteins, including *SCC2*, *WAPL2*, and *Shugoshin*, help establish the meiotic axis and form the physical scaffold necessary for proper homolog alignment. Second, DNA methylation and heterochromatin‐associated proteins, such as *DDM1* and *SUVH2*, contribute to shaping the chromatin landscape to regulate the formation of DSBs, which are essential for recombination. Third, MMR proteins, including *MutS* and *PMS1*, destabilize mismatched recombination intermediates to favor homologous over homeologous pairing. Finally, spindle checkpoint components, such as *MPS1‐2*, ensure accurate chromosome orientation and segregation during meiosis.

### Barriers to autopolyploids as progenitors of allopolyploids

The limited contribution of autopolyploids to the origin of allopolyploids raises questions about their suitability as allopolyploid progenitors. Despite harboring alleles pre‐adapted to polyploidy, we propose that autopolyploids may be constrained in two key ways. First, autopolyploids with four homologous chromosome copies can accumulate deleterious mutations due to relaxed purifying selection (Baduel *et al*., 2019; Bao *et al*., 2022). When an autopolyploid gamete contributes to the origin of an allopolyploid, these mutations may become exposed and, if not fully compensated by a homeolog, could lead to reduced fitness and increased extinction risk. Second, autopolyploids like *A. arenosa* exhibit altered meiotic crossover patterns (Bomblies *et al*., 2016). Whether these altered crossover patterns are beneficial or reversible or could be disruptive for homeolog segregation in allopolyploids remains unknown, but they may further reduce their viability as allopolyploid parents.

### Homeolog compensation and early stages of rediploidization

We find evidence of relaxed purifying selection in *A. suecica*, consistent with the idea that polyploidy confers increased mutational robustness through genetic redundancy. Previous studies have shown that diploid *A. thaliana* is more sensitive to mutagenesis than both natural and synthetic *A. suecica* (Spoelhof *et al*., 2021), supporting the claim that *A. suecica* has enhanced mutational robustness compared with its diploid parents. Despite the evidence of relaxed purifying selection, *A. suecica* has accumulated relatively few genes with a *de novo* LoF mutation, suggesting that selection has still remained sufficiently strong to limit the accumulation of new LoF variants.

We observed bias in *A*. *suecica* toward LoF mutations in the *A. arenosa* sub‐genome; however, most of these LoF mutations also segregate in *A. arenosa* and are likely inherited. This higher number of inherited mutations from the *A. arenosa* sub‐genome is due to the higher genetic diversity and outcrossing nature of *A. arenosa*, which allows for better masking of deleterious mutations compared with the highly homozygous and predominantly selfing *A. thaliana*. *De novo* LoF mutations did not show sub‐genome bias, consistent with our previous study, showing the absence of sub‐genome dominance on the transcriptional level (Burns *et al*., 2021).

Our analysis of LoF mutations in *A. suecica* reveals a pattern of compensation between homeologs, whereby the retention of at least one functional copy mitigates the impact of deleterious mutations. Specifically, when a LoF mutation arises in one homeolog, the other tends to remain intact more often than expected by chance. We find that these retained homeologs are under purifying selection, suggesting a selective pressure to preserve essential gene functions. This pattern aligns with findings in yeast, where duplicate genes can enhance mutational robustness but are often rapidly inactivated by LoF mutations, rather than undergoing gradual subfunctionalization (Mihajlovic *et al*., 2024). The homeolog compensation pattern is symmetric across sub‐genomes; we find no bias in the accumulation of *de novo* LoF mutations or in the representation of dosage‐sensitive single‐copy genes. These patterns suggest that, shortly after allopolyploidization, mutations occur at random across sub‐genomes, with selection acting *post hoc* to retain at least one functional copy per gene pair. While relaxed purifying selection allows LoF mutations to accrue, the compensatory retention of one intact copy buffers against gene function loss. This buffering, leveraging both standing and *de novo* variation, likely facilitates early genome stabilization in polyploids and helps maintain overall functional integrity. Yet, this same genetic robustness may come at a cost: By masking the effects of deleterious mutations, homeolog compensation facilitates their persistence, setting the stage for gene loss to occur over time. We propose that homeolog compensatory dynamics mark the very beginning of rediploidization, a process by which the initial robustness of a polyploid genome ultimately shapes its return to a diploid‐like state.

## Competing interests

None declared.

## Author contributions

RB and PYN conceived the study. Herbarium samples were acquired by PYN, and genomic DNA was extracted by RB. RB, PYN, AK, AG, UKK and ADS analyzed the data. RB, PYN, FK and ADS wrote the paper.

## Disclaimer

The New Phytologist Foundation remains neutral with regard to jurisdictional claims in maps and in any institutional affiliations.

## Supporting information


**Dataset S1** Median nucleotide diversity of 163 *A. arenosa* individuals and 966 *A. thaliana* accession to *A. suecica*.

**Dataset S2** Genes identified under positive selection in *A. suecica* using McDonald–Kreitman (MK test) and homeologs with loss‐of‐function mutations.

**Fig. S1** SNP sharing and nucleotide diversity (π) in *A. suecica*.
**Fig. S2** The geographic origin of *A. suecica*.
**Fig. S3** In the *A. thaliana* subgenome (Chr1 to 5), 35 genes, and in the *A. arenosa* subgenome (Chr 6 to 13), 585 genes show signatures of positive selection.
**Fig. S4** GO analysis of selection scan genes in *A. suecica* GO enrichment for the 35 genes on the *A. thaliana* subgenome and the 585 genes on the *A. arenosa* subgenome.
**Fig. S5** Overlap of genes in selection scans and homeologous gene pairs carrying one LoF mutation.
**Fig. S6**. Subgenome expression bias toward intact homeolog.
**Fig. S7** Lack of enrichment for LoF mutations in single‐copy genes in *A. suecica*.
**Notes S1** The geographic origins of *A. suecica*.
Please note: Wiley is not responsible for the content or functionality of any Supporting Information supplied by the authors. Any queries (other than missing material) should be directed to the *New Phytologist* Central Office.

## Data Availability

Raw fastq files of the Illumina sequenced herbarium samples for five tetraploid *A. arenosa* and one *A. suecica* generated in this study were deposited to the European Nucleotide Archive (ENA) under the project number PRJEB79527. Custom scripts used in this study are available at https://github.com/burnsro/ArabidopsisSuecica/tree/main/origin.
