# Supplementary material for: Diploid origins and early genome stabilization in the allotetraploid Arabidopsis suecica
Source: New Phytol. 2025 Nov 5;249(1):524–38. doi: 10.1111/nph.70689 (PMC12676072; doi:10.1111/nph.70689)
Supplement: Supplementary file 3 — Fig. S1 SNP sharing and nucleotide diversity (π) in A. suecica. Fig. S2 The geographic origin of A. suecica. Fig. S3 In the A. thaliana subgenome (Chr1 to 5), 35 genes, and in the A. arenosa subgenome (Chr 6 to 13), 585 genes show signatures of positive selection. Fig. S4 GO analysis of selection scan genes in A. suecica GO enrichment for the 35 genes on the A. thaliana subgenome and the 585 genes on the A. arenosa subgenome. Fig. S5 Overlap of genes in selection scans and homeologous gene pairs carrying one LoF mutation. Fig. S6. Subgenome expression bias toward intact homeolog. Fig. S7 Lack of enrichment for LoF mutations in single‐copy genes in A. suecica. Notes S1 The geographic origins of A. suecica. Please note: Wiley is not responsible for the content or functionality of any Supporting Information supplied by the authors. Any queries (other than missing material) should be directed to the New Phytologist Central Office. [file NPH-249-524-s002.docx]

#### New Phytologist Supporting Information

#### Article title: Diploid origins and early genome stabilization in the allotetraploid Arabidopsis suecica

#### Authors: Robin Burns, Anna Glushkevich, Aboli Kulkarni, Uliana K Kolesnikova, Filip Kolář, Alison Dawn Scott, Polina Yu. Novikova

#### Article acceptance date: 6 October 2025

# Supplemental Figures


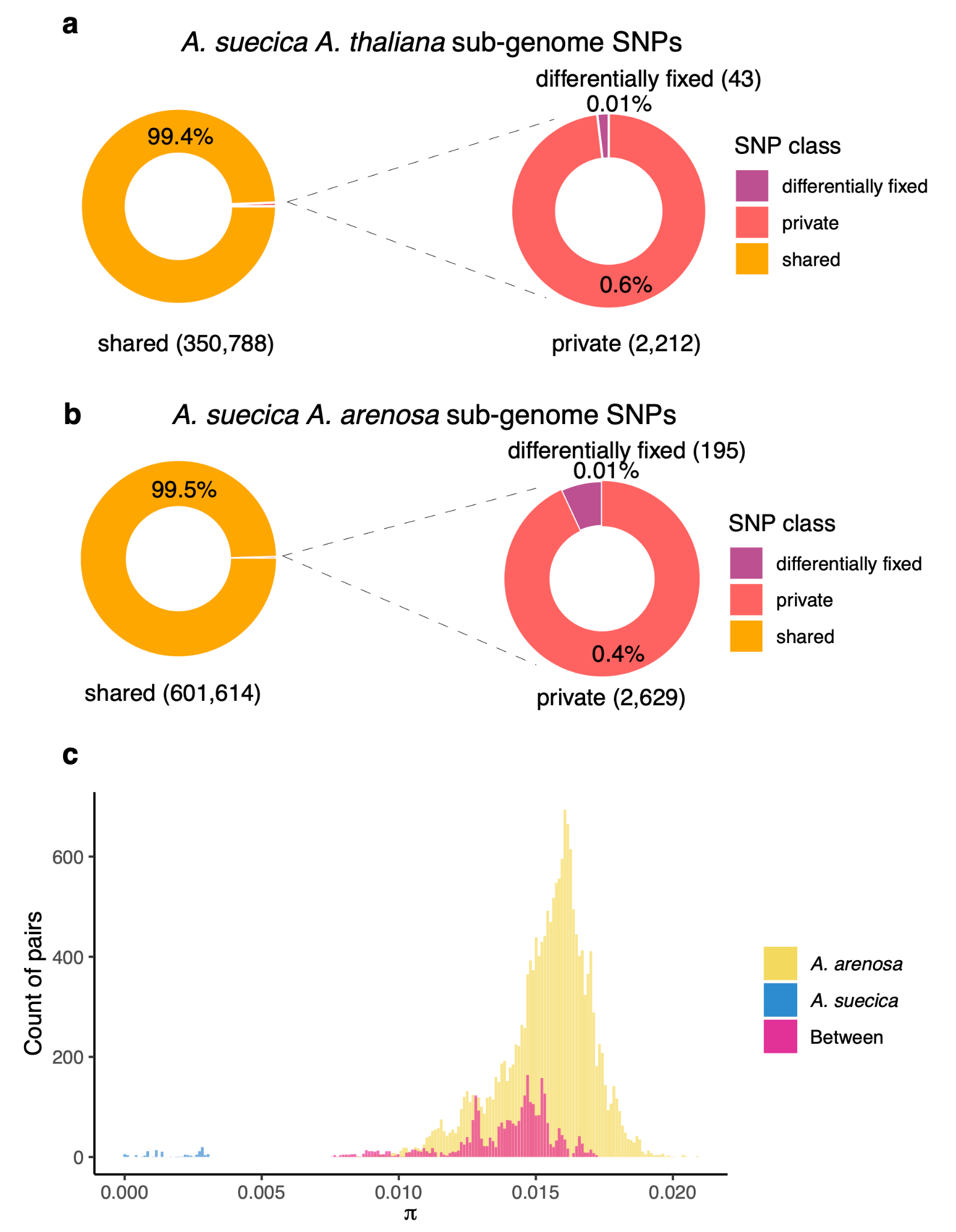


**Supplementary Figure 1: SNP Sharing and Nucleotide Diversity (π) in *A. suecica***

**a, b** Over 99% of *A. suecica* SNPs are shared with progenitor species (*A. thaliana* and *A. arenosa*), indicating contributions from multiple individuals of both progenitors. **c** Nucleotide diversity (π) is significantly lower in *A. suecica* (blue) compared to *A. arenosa* (yellow) due to the allopolyploidy bottleneck and self-compatibility. In contrast, the obligate outcrosser *A. arenosa* displays greater diversity within species than between species (pink).


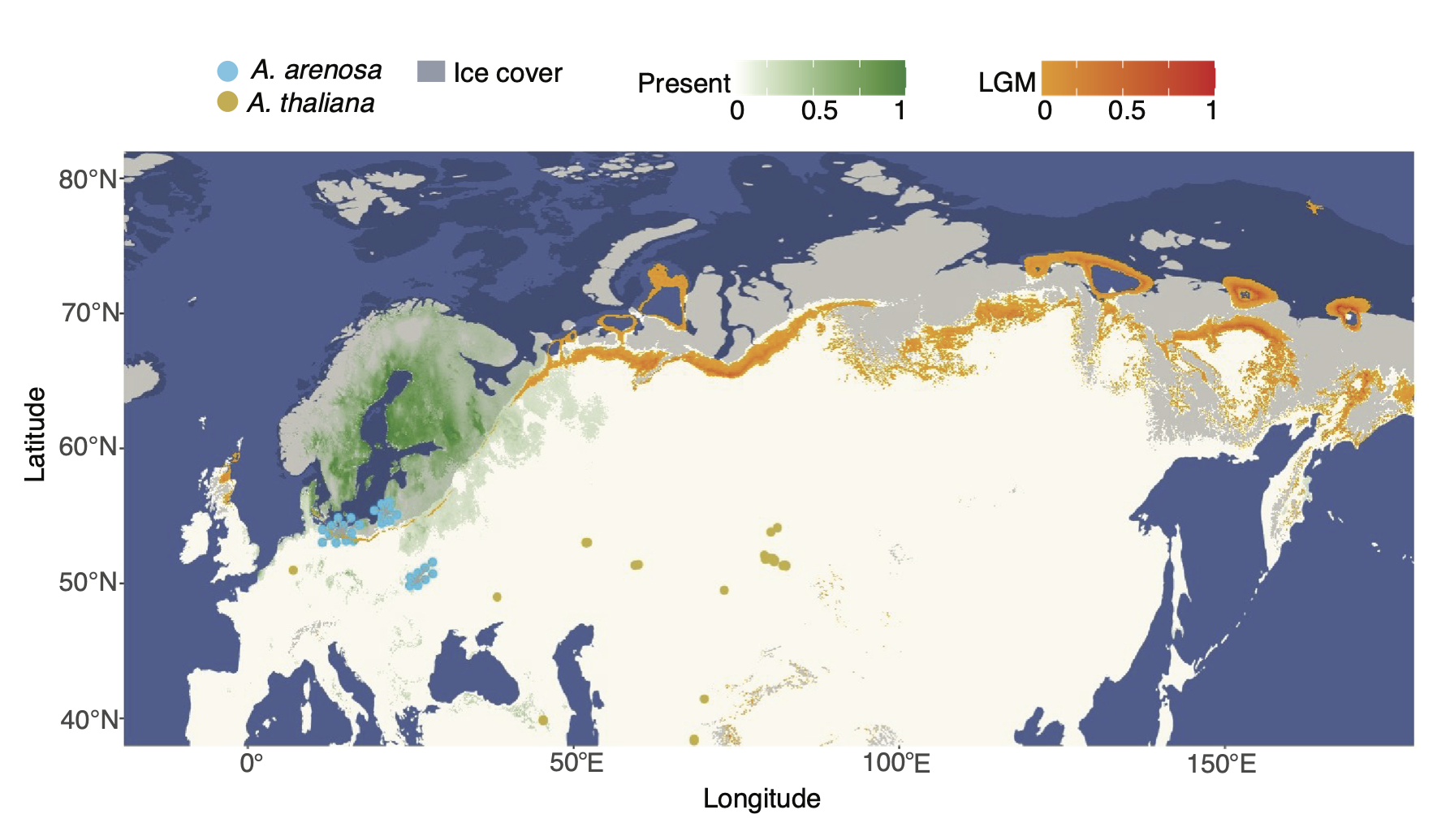


**Supplementary Figure 2: The geographic origin of *A. suecica*.** Niche modeling of *A. suecica* shows probability of occurrence for the current climate (green) and the LGM (orange). Closest parental populations include Southern Baltic and Ukrainian diploid *A. arenosa*, and the top 5% of *A. thaliana* accessions genetically closest to *A. suecica*.


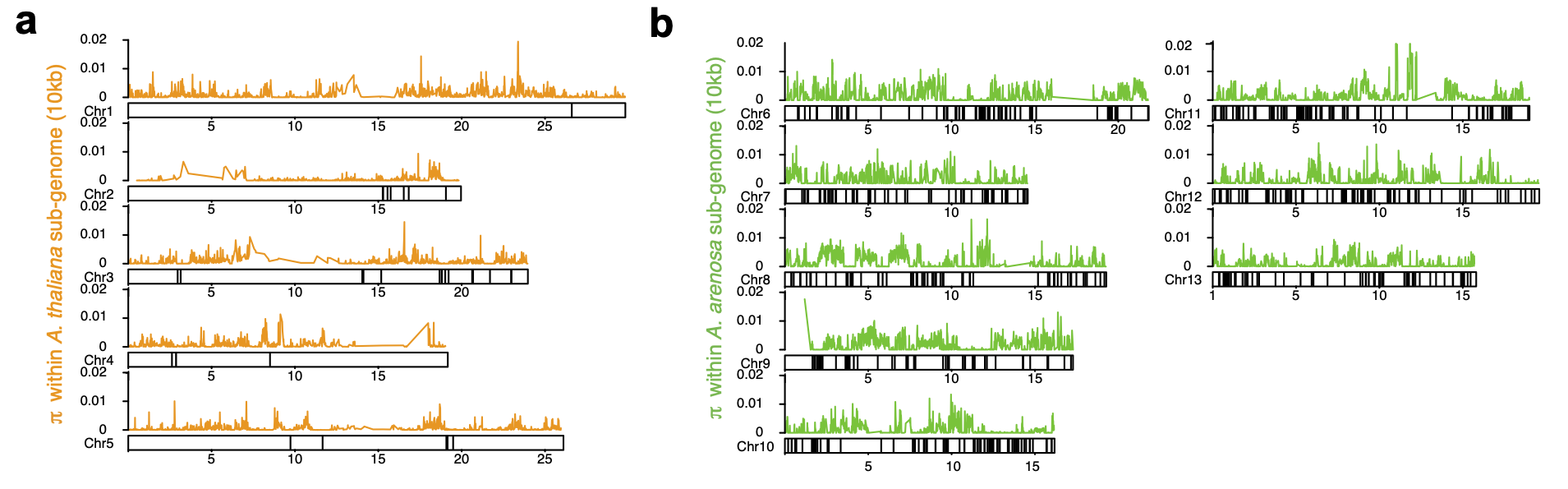


**Supplementary Figure 3 a** In the *A. thaliana* sub-genome (Chr1 to 5), 35 genes, and **b** in the *A. arenosa* sub-genome (Chr 6 to 13), 585 genes show signatures of positive selection. These genes are found outside of the areas of low nucleotide diversity (π) within *A. suecica*, meaning a founder effect or population bottleneck is not the likely explanation for the signature of positive selection.


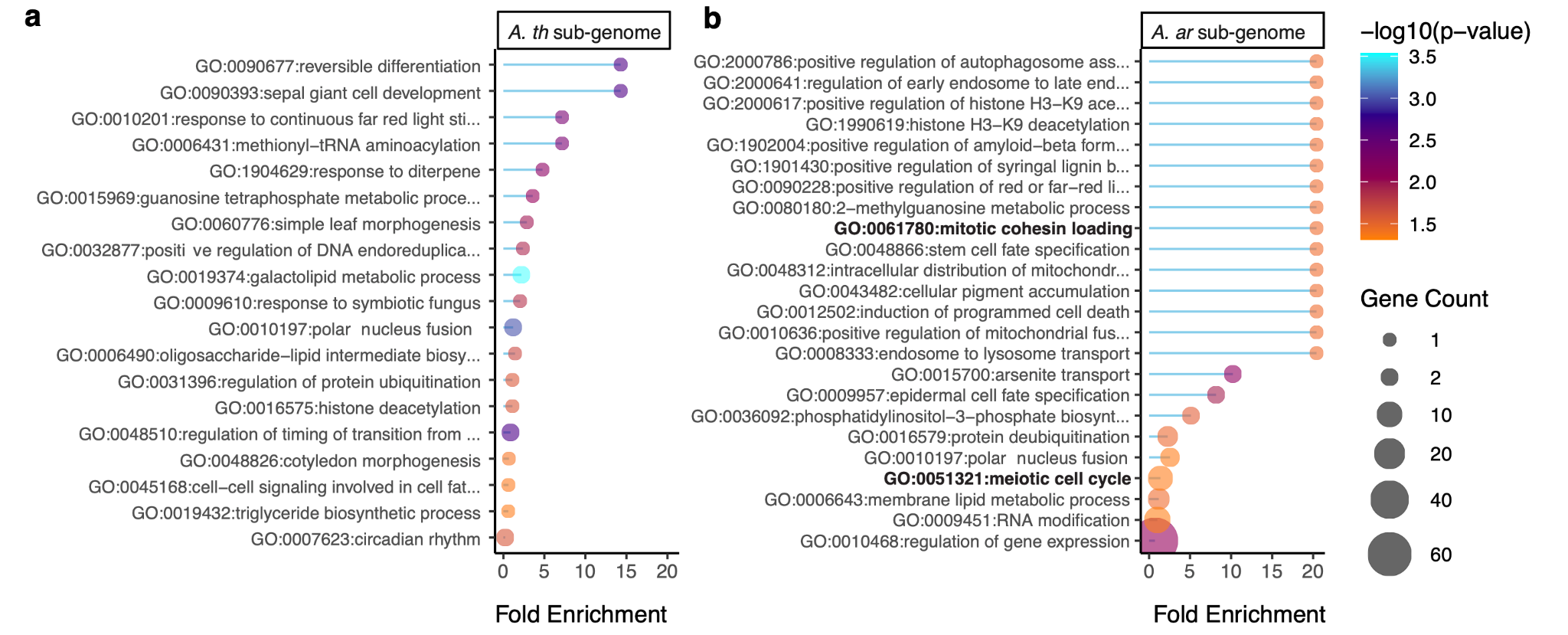


**Supplementary Figure 4. GO analysis of selection scan genes in *A. suecica*** GO enrichment for the **a** 35 genes on the A. thaliana sub-genome and **b** the 585 genes on the A. arenosa sub-genome. GO terms mentioned in the text are highlighted in bold.


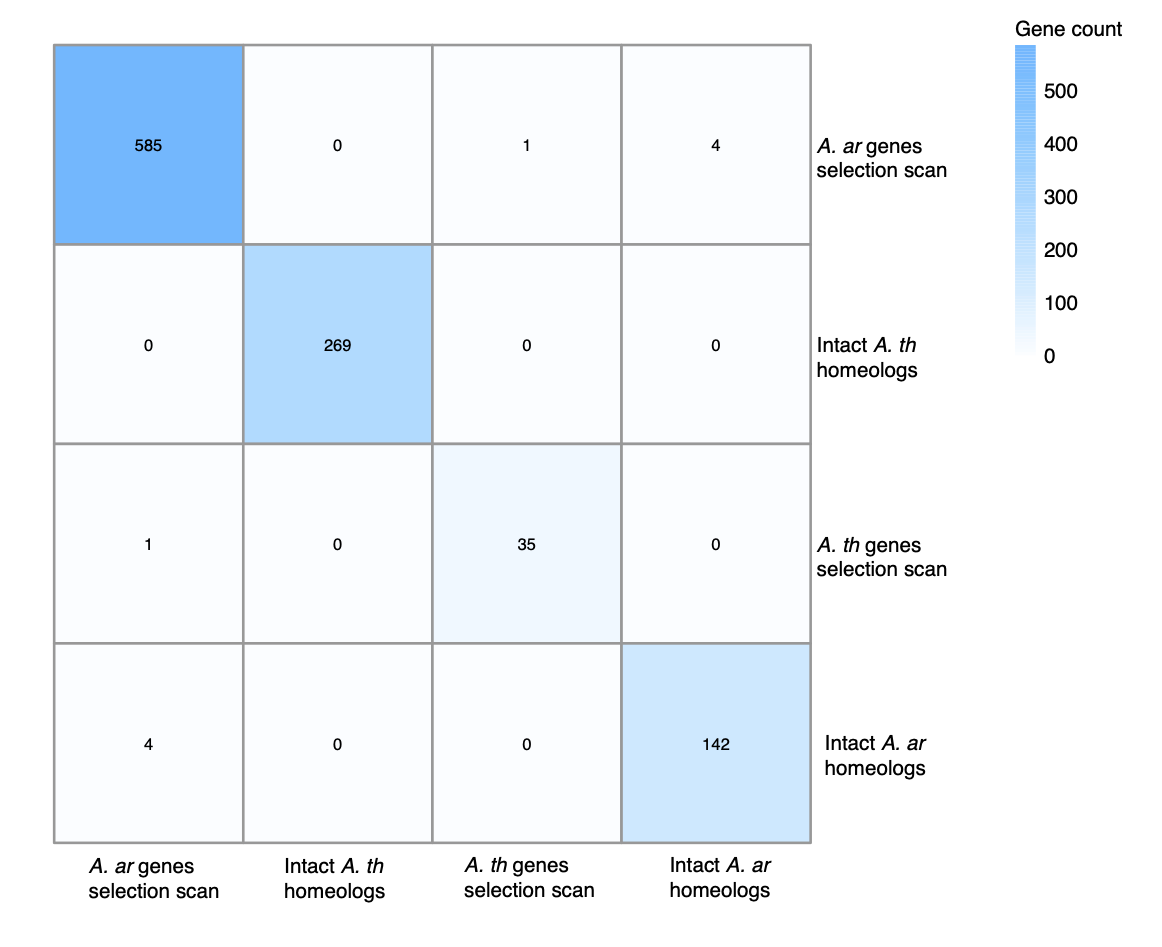


**Supplementary Figure 5 Overlap of genes in selection scans and homeologous gene pairs carrying one LoF mutation.** Minimal overlap was observed between genes from selection scans and homeologous gene pairs with one intact copy and one carrying a LoF mutation, indicating that LoF on one homeolog is not driving the likely adaptive genetic changes on the other.

#
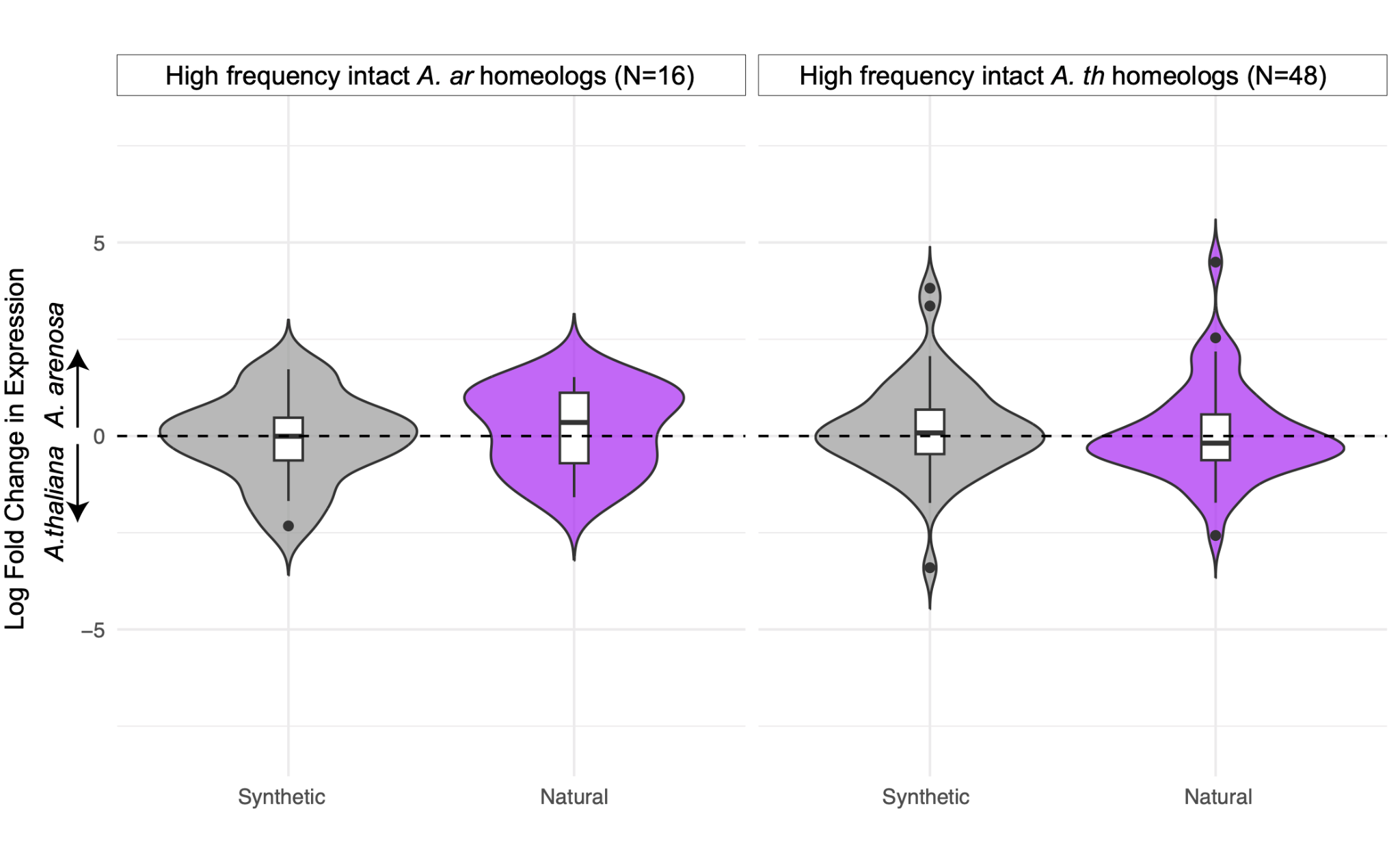


**Supplementary Figure 6. Sub-genome expression bias towards intact homeolog*.*** Violin plots show the log_2_ fold change in gene expression between homeologs in synthetic versus natural *A. suecica* accessions. Expression bias is plotted for genes where one homeolog exhibits a high-frequency loss-of-function mutation (≥60% in the population) while the homeolog either on the *A. arenosa* sub-genome (left panel, N = 16) or in the *A. thaliana* sub-genome (right panel, N = 48) remains intact. Each violin represents the distribution of expression bias for either synthetic (gray) or natural (purple) *A. suecica*. Positive values indicate an expression bias towards the *A. arenosa* homeolog and negative values indicated an expression bias towards the *A. thaliana* homeolog. A log fold change in gene expression of zero (dashed line) indicates balanced expression between homeologs. Expression in natural *A. suecica* is biased toward the intact homeolog, consistent with purifying selection maintaining expression of the functional copy. This expression bias is absent in synthetic *A. suecica*, indicating that it is not an inherited regulatory pattern but rather an evolved expression change due to the presence of a nonfunctional allele in the other homeolog.


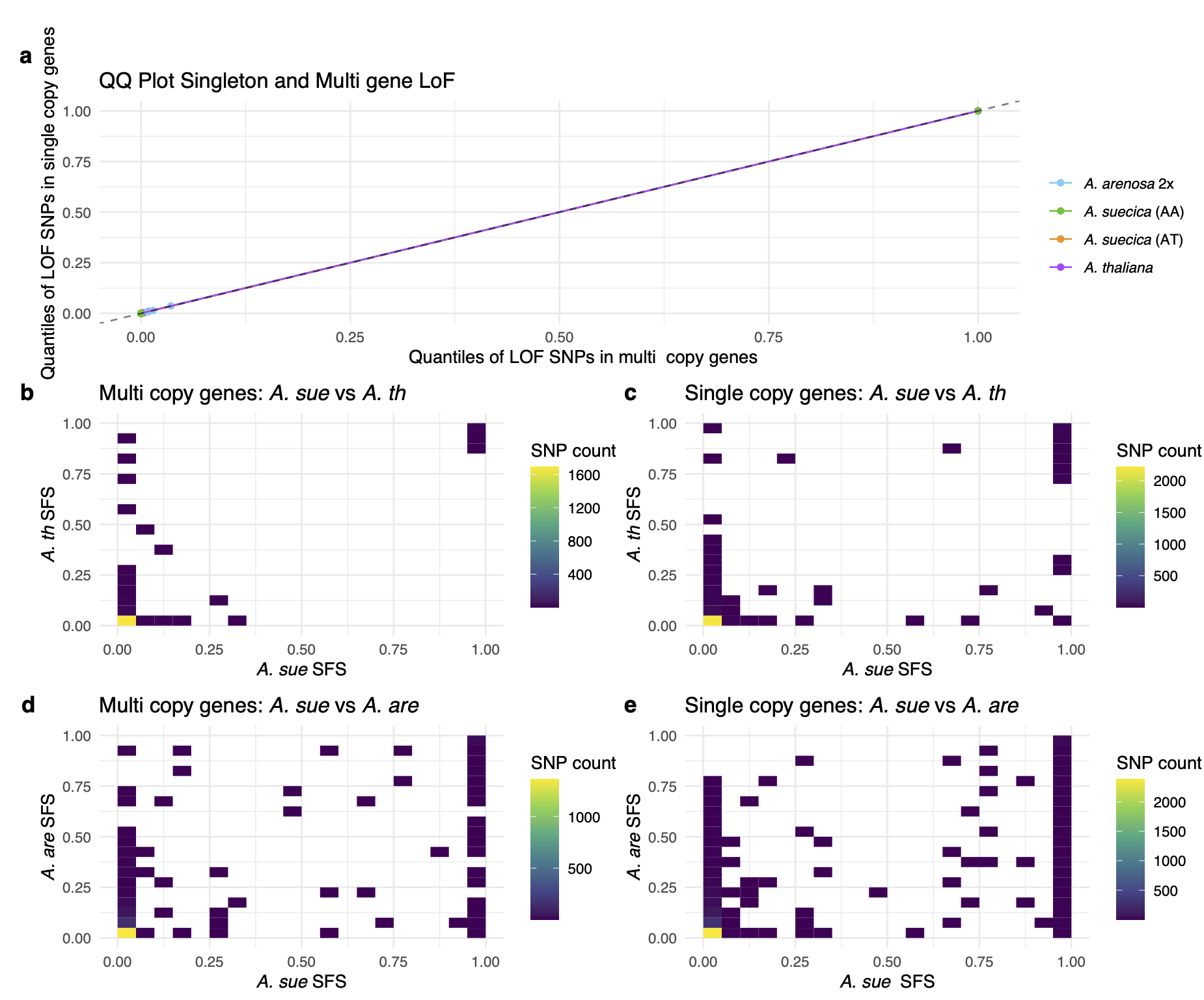


**Supplementary Figure 7. Lack of enrichment for LoF mutations in single-copy genes in *A. suecica.* a** QQ plots comparing the population frequencies of LoF SNPs in single-copy genes versus multi-copy genes in *A. suecica*. No significant increase in the population frequency of LoF mutations is observed in *A. suecica* for single-copy genes, indicating that both single- and multi-copy genes are under similar selective pressures. **b-e** Joint allele frequency spectra for LoF SNPs, comparing *A. suecica* and the parental species for multi-copy and single-copy genes. The data show an enrichment of singleton SNPs for all species and gene copy status, consistent with strong purifying selection acting on these deleterious mutations.

#

#

# Supplementary Note 1

## The geographic origins of *A. suecica*

The origin of *A. suecica* is estimated to be around 16,000 years ago[[56]](https://paperpile.com/c/4pQ10x/rD8s), when Fennoscandia, where *A. suecica* is currently distributed [50], was sheathed in ice. The genetically closest *A. thaliana* accessions to *A. suecica* are located in Central Eurasia (Figure 1A) and the closest *A. arenosa* individuals are located in the Southern Baltics (Figure 1C). This striking geographic separation between the most closely related parental populations raises the question of where, geographically and ecologically, hybridization could have occurred between *A. thaliana* and *A. arenosa*.

To explore whether there was any potential climatic or geographic overlap between these parental lineages at the time of *A. suecica*’s origin, we performed ecological niche modeling. We used the occurrence data for *A. suecica* and corresponding climatic variables to predict the potential distribution of the species across Eurasia (see Methods). We included all of Eurasia to fully capture the geographic distribution of both parental species*.* The model showed that the present climate niche aligns well with the current distribution of *A. suecica*, which is restricted to Fennoscandia (Supplementary Figure 7, green layer).

Next, we projected the current niche of *A. suecica* onto the climate of the Last Glacial Maximum (LGM), the estimated time of the origin for *A. suecica[[56]](https://paperpile.com/c/4pQ10x/rD8s)*, assuming that the niche remained similar. The projection resulted in a very narrow distribution following the edge of the ice sheet (Supplementary Figure 7, orange layer), which could be suitable for *A. suecica.* This projected niche partially overlaps with the current distribution of the diploid *A. arenosa* that is genetically closest to *A. suecica,* near the southern Baltic Sea coast (Figure 1C, red circles). While the location of the genetically closest *A. thaliana* accessions is far from the Baltics (Figure 1A, red circles), several *A. thaliana* accessions, included in the top 5% of the genetically closest to *A. suecica*, are located relatively near the southern Baltic region (Supplementary Figure 7, light brown circles).

Based on these findings we suggest that the likely geographic origin for *A. suecica* was near the southern Baltic coast during the last ice age, after which *A. suecica* migrated to Fennoscandia. The movement of *A. thaliana* eastwards is also consistent with the demographic post-glacial history of the species[[116]](https://paperpile.com/c/4pQ10x/AxXx).
